# Epigenetic Control of Mitochondrial Function in the Vasculature

**DOI:** 10.3389/fcvm.2020.00028

**Published:** 2020-03-04

**Authors:** Shafeeq A. Mohammed, Samuele Ambrosini, Thomas Lüscher, Francesco Paneni, Sarah Costantino

**Affiliations:** ^1^Center for Molecular Cardiology, University of Zürich, Zurich, Switzerland; ^2^Research, Education and Development, Royal Brompton and Harefield Hospital Trust and Imperial College, London, United Kingdom; ^3^Department of Cardiology, University Heart Center, University Hospital Zurich, Zurich, Switzerland; ^4^Department of Research and Education, University Hospital Zurich, Zurich, Switzerland

**Keywords:** epigenetics, mitochondria, vascular disease, oxidative stress, endothelial function

## Abstract

The molecular signatures of epigenetic regulation and chromatin architecture are emerging as pivotal regulators of mitochondrial function. Recent studies unveiled a complex intersection among environmental factors, epigenetic signals, and mitochondrial metabolism, ultimately leading to alterations of vascular phenotype and increased cardiovascular risk. Changing environmental conditions over the lifetime induce covalent and post-translational chemical modification of the chromatin template which sensitize the genome to establish new transcriptional programs and, hence, diverse functional states. On the other hand, metabolic alterations occurring in mitochondria affect the availability of substrates for chromatin-modifying enzymes, thus leading to maladaptive epigenetic signatures altering chromatin accessibility and gene transcription. Indeed, several components of the epigenetic machinery require intermediates of cellular metabolism (ATP, AcCoA, NADH, α-ketoglutarate) for enzymatic function. In the present review, we describe the emerging role of epigenetic modifications as fine tuners of gene transcription in mitochondrial dysfunction and vascular disease. Specifically, the following aspects are described in detail: (i) mitochondria and vascular function, (ii) mitochondrial ROS, (iii) epigenetic regulation of mitochondrial function; (iv) the role of mitochondrial metabolites as key effectors for chromatin-modifying enzymes; (v) epigenetic therapies. Understanding epigenetic routes may pave the way for new approaches to develop personalized therapies to prevent mitochondrial insufficiency and its complications.

## Mitochondria and Vascular Function

**Figure 1:**
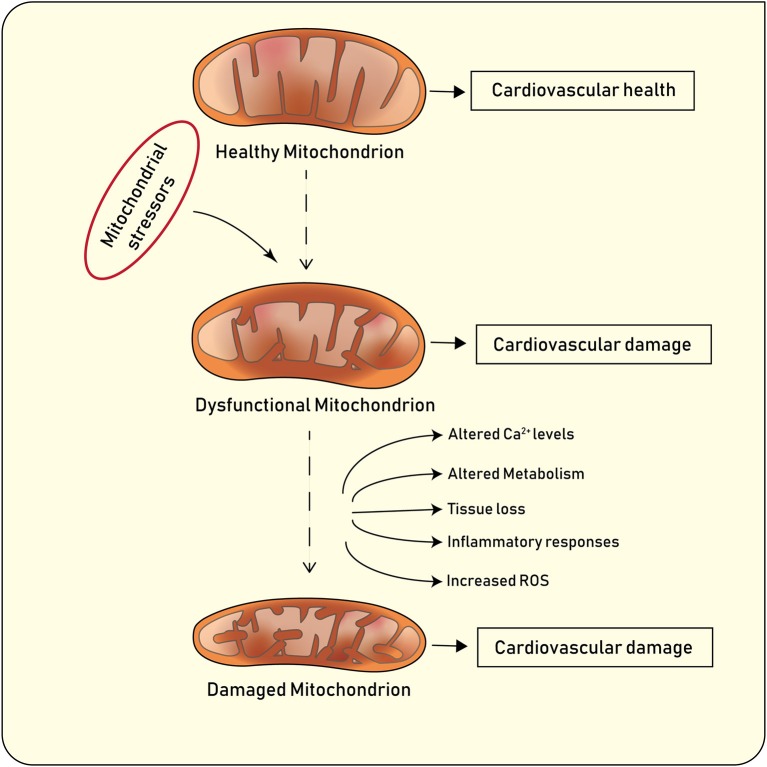
Main features of healthy and diseased mitochondria, and implications for cardiovascular disease.

Mitochondria, defined as semi-autonomous, membrane-bound organelle localized in the cytoplasm of eukaryotic cells, are emerging as a pivotal player in health, disease, and aging by regulating reactive oxygen species (ROS) production and contributing to retrograde redox signalling from the organelle to the cytosol and nucleus (Figure 1) (1, 2). Mitochondria play an important role in the overall cellular network formed by metabolic signalling and epigenetic pathways. Indeed, mitochondria drive catabolic and anabolic reactions supplying energy and metabolites with biosynthetic and signalling roles (3). They also maintain a bidirectional signalling crosstalk with the nucleus that generates reciprocal activation-repression patterns of gene expression (3–5). Finally, mitochondria can determine apoptotic and necrotic cell death mediated by Ca^2+^ overload and opening of the permeability transition pore (PTP) (6, 7).

Under physiological conditions, mitochondria undergo highly coordinated cycles of fission (division of a single organelle into two or more independent structures) or fusion (the opposing reaction) (8). Fission and fusion are active processes which require many specialized proteins, including mechanical enzymes that physically alter mitochondrial membranes, and adaptor proteins that regulate the interaction of these mechanical proteins with organelles. The balance between these two processes regulates the overall morphology of mitochondria within any given cell (8–10). The content of mitochondria in the cytoplasm of eukaryotic cells depend on two major processes known as mitochondrial biogenesis and mitophagy (11). Mitochondrial biogenesis is an intricate and not fully understood process which leads to an increased mitochondrial mass mainly via replication of mitochondrial DNA (mtDNA) and expression of nuclear and mitochondrial genes (12). PGC-1α (Peroxisome proliferator-activated receptor gamma coactivator−1α) plays a prominent role in mitochondrial biogenesis by activating the nuclear respiratory factor (Nrf)-1 and −2 to promote the expression nuclear genes. PGC-1α also activates transcription factors A and B which regulate the expression of mitochondrial genes (13, 14). Following mitochondrial damage, the organelles are being selectively degraded according to a well-known biological process called mitophagy, which promotes organelle turnover while preventing accumulation of dysfunctional mitochondria (Figure 1) (11). In addition to the selective removal of damaged mitochondria, mitophagy is also required to adjust mitochondrial numbers to changing cellular metabolic needs, for steady-state mitochondrial turnover, and during certain cellular developmental stages, such as during cellular differentiation of red blood cells (10). Mitochondrial content may vary based on the cell type and its function. For example, in endothelial cells mitochondria occupy around 6% of cytoplasm whereas in cardiomyocytes this reaches 32% (15). Notably, the blood brain barrier which consist of highly active endothelial cells has higher mitochondrial content as compared with endothelial cells present in capillary beds (15). Mitochondria play a pivotal role in endothelial cells. Several biological processes including mitochondrial biogenesis, fission and fusion as well as mitophagy, have shown to clearly affect endothelial cell function and metabolism. Several stimuli including hypoxia, calorie restriction or exercise induce mitochondrial biogenesis in endothelial cells by increasing the expression of the peroxisome proliferator-activated receptor-γ coactivator-1α (PGC-1α). Induction of PGC-1α is associated with a favorable transcriptional profile which protects endothelial cells from oxidative damage and apoptosis (16). In line with this notion, endothelial-specific overexpression of PGC-1α protects against angiotensin II–induced hypertension (17). By contrast, loss of endothelial PGC-1α impairs endothelial NO bioactivity eventually leading to endothelial dysfunction (18). Alterations of mitochondrial dynamics also contribute to endothelial cell phenotype. Endothelial cells from patients with diabetes display mitochondrial fragmentation and increased expression of fission-1 protein (Fis1) and dynamin-related protein-1 (Drp1). Of note, *in vitro* experiments showed that gene silencing Fis1 or Drp1 expression blunted hyperglycemia-induced alterations in mitochondrial networks, ROS production, endothelial nitric oxide synthase activation, and cGMP production (19). Alterations of mitophagy as the result of disturbed Ucp2/PTEN signaling were also associated with inadequate mitochondrial biosynthesis and increased apoptosis in endothelium (20). Altered mitochondrial clearance may also contribute to age-dependent endothelial dysfunction. Indeed, senescent cells display altered mitochondrial dynamics and loss of membrane potential (21). Interestingly enough, overexpression of proteins involved in the autophagosome formation (ATG5 and ATG12) was associated with improved mitochondrial performance, as evidenced by higher membrane potential, increased ATP production, and decreased damage to mtDNA (22, 23).

## Mitochondrial ROS

Although several cytosolic enzymes (i.e., NADPH, cyclooxygenases, and xanthine oxidase) are implicated in redox balance, ROS generated from mitochondrial oxidative phosphorylation represent the most important source of oxidative stress in vascular cells (i.e., endothelial cells) (24, 25).

Mitochondrial ROS are responsible for peroxidation of polyunsaturated fatty acids (PUFAs) present in the cellular membrane as well as DNA (causing single and double strand breaks) and protein damage via oxidation of sulfhydryl and aldehyde groups, protein-protein interactions and fragmentation (26). In addition, damage of mtDNA may lead to decreased expression of electron transport chain components or expression of defective components that produce more ROS, thus creating a detrimental vicious cycle. mtDNA disruption also correlates with the extent of atherosclerosis in mouse models and human tissues. Despite the highly efficient chemical reduction of O_2_ through cytochrome *c* oxidase, mitochondria still generate significant levels of ROS (27). Cellular and mitochondrial physiological levels of ROS are reached when production and scavenging are balanced (28). Mitochondrial dysfunction is believed to play an important role in a variety of diseases including diabetes, obesity, dyslipidaemia, hypertension, arrhythmias, and sudden cardiac death (29–31).

In the setting of cardiovascular risk factors, namely hyperglycemia, mitochondrial ROS can be regarded as an upstream biochemical event responsible for the activation of pro-inflammatory pathways (i.e., NF-kB), protein kinase C as well as advanced glycation end products (AGEs) (32). An increasing body of evidence has contributed to unveil different sources of mitochondrial ROS in endothelial cells. Studies in isolated mitochondria have shown that superoxide anion formation at complexes I and III accounts for 0.1-2% of the total (33). In addition to complexes I and III, the nicotinamide adenine dinucleotide phosphate oxidase (NOX) 4—a ROS-generating enzyme involved in endothelial cell senescence, migration, angiogenesis, and adaptive responses to hypoxia—is highly expressed in vascular cells and has been localized to mitochondria (34). Moreover, the monoamine oxidase (MAO) family of enzymes—which is found in the outer mitochondrial membrane—generates hydrogen peroxide (H_2_O_2_) during catabolism of catecholamines and has been implicated in maladaptive cellular hypertrophy and apoptosis (35). MAO-A-induced ROS are involved in serotonin-induced vasoconstriction in vascular smooth muscle cells (36). Although endothelial cells are known to express MAO, its importance for endothelial function is poorly understood (37). The mitochondrial adaptor protein p66^Shc^ was recently shown to be causally involved in mitochondrial ROS generation and cellular death. In conditions of cellular stress, p66^Shc^ is phosphorylated at ser36 by protein kinase C beta2 (PKCβ2) and translocates to the mitochondria where it oxidizes cytochrome *c*, leading to accumulation of H_2_O_2_, PTP opening, and release of solutes and proapoptotic signals (38). The causal role of p66^Shc^ in vascular disease is supported by the notion that its genetic deletion or gene silencing prevents age and hyperglycemia-induced endothelial dysfunction in mice (39–41). The prolyl-isomerase 1 (Pin1), which regulates p66^Shc^ translocation to the mitochondria, has also shown to be causally implicated in the regulation of mitochondrial oxidative stress and integrity in experimental models of diabetes (42, 43). The mitochondrial ATP-sensitive potassium channel (mitoK_ATP_) was also recently discovered as a potential source of mitochondrial ROS in cardiac myocytes (44). Although the exact mechanism of action remains elusive, mitoK_ATP_ seems to act as an uncoupling agent by reducing membrane potential and mitochondrial calcium. Pharmacological inhibition of mitoK_ATP_ was found to improve endothelial function and to prevent ischemia-induced cellular apoptosis (44). Several antioxidant enzymes play a pivotal role in maintaining redox balance in mitochondria. Manganese superoxide dismutase (MnSOD) represents one of the first line defense against accumulation of mitochondrial superoxide. MnSOD is located in the mitochondrial matrix and catalyzes the conversion of superoxide anion to hydrogen peroxide (45). Loss of MnSOD in mice leads to impaired endothelium-dependent vasodilation, suggesting its role in regulating vascular function. In addition, *ApoE*^−/−^ *MnSOD*^+/−^ mice display early mtDNA damage and accelerated atherosclerosis when compared to control animals (46). Levels of H_2_O_2_ are regulated by glutathione peroxidase-1, thioredoxin-2, peroxiridoxin-3, and glutaredoxin-2 (47). As noted, increased expression of these enzymes is signaled by AMPK and PGC-1α in response to H_2_O_2_ and other free radicals in endothelial cells (48). Studies in experimental models have shown that reduced expression of mitochondrial antioxidant enzymes can induce mitochondrial damage, endothelial dysfunction, and atherogenesis (45, 46). Conversely, overexpression of these proteins is protective against the development of vascular disease (49).

Although the role of mitochondrial ROS in vascular damage is well-established, only few studies have explored the specific contribution of mitochondria-derived ROS in the pathophysiology of endothelial dysfunction in humans. Mitochondrial ROS production and membrane hyperpolarization are significantly altered in visceral fat arteries and peripheral blood mononuclear cells isolated from patients with obesity and type 2 diabetes (50, 51). Furthermore, impaired endothelium-dependent vasodilation in freshly isolated arterioles from diabetic individuals is reversed by mild membrane depolarization or mitochondria-targeted antioxidants (50).

## Epigenetic Regulation of Mitochondrial Function

**Figure 2:**
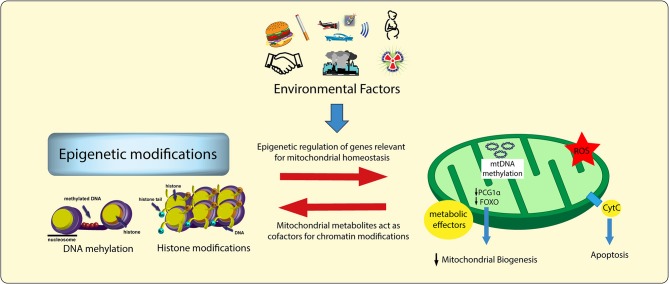
Environmental factors, chromatin modifications, and mitochondrial damage. Environmental factors lead to specific epigenetic signatures as well as to alterations of mitochondrial intermediate metabolites (i.e., acetyl-CoA, FAD+, NAD+). These two processes influence each other, thus leading to a vicious cycle responsible for adverse chromatin modifications, maladaptive transcriptional programs, and vascular dysfunction. ROS, reactive oxygen species.

Recent evidence indicates that epigenetic changes, defined as plastic modifications of DNA/histone complexes, are heavily implicated in the regulation of mitochondrial and vascular function (52, 53). Studies conducted over the last few years have unmasked a complex intersection among environmental factors, mitochondrial metabolism, epigenetic signals and transcriptional programs (54, 55). Epigenetic changes acquired during the life time may derail the expression of genes involved in mitochondrial homeostasis (52). On the other hand, metabolic alterations occurring in mitochondria may affect the availability of substrates for chromatin-modifying enzymes, thus leading to maladaptive epigenetic signatures altering chromatin accessibility and, hence, gene transcription (Figure 2) (54). Indeed, the availability of some intermediate mitochondrial metabolites (ATP, AcCoA, NADH, α-ketoglutarate) has shown to foster different patterns of epigenetic modifications. For examples, iron, α-ketoglutarate (α-KG) and O_2_ are needed both for histone demethylation—catalysed by iron-containing jumonji-domain (jmjC) demethylases (56)—as well as for DNA demethylation of 5-methylcytosine—catalysed by the ten-eleven translocation family of dioxygenases (TET) (57). Therefore, mitochondrial sensitivity determined by environmental factors and lifestyle changes (sedentarism, physical activity, overnutrition, balanced nutrition) will favor, or prevent, the effects of metabolic disorders.

## Classification of Epigenetic Changes

Epigenetic mechanisms can be divided into three main categories: (i) chemical modifications of DNA (i.e., methylation); (ii) post-translational modifications of histone tails; (iii) regulation of gene expression by non-coding RNAs [i.e., microRNAs, long non-coding RNAs (lncRNAs)] (58). In the present review, we will focus on the modifications of DNA/histone complexes and their impact on mitochondrial integrity and functionality.

### DNA Methylation

Methylation of DNA mainly takes place at the level of CpG regions of gene promoters through the attachment of methyl group (CH3) from S-adenosyl methionine (SAM) to the C5 position in the cytosine-paired-with-guanine (CpG) dinucleotide sequences (59). CpG sequences are generally located into promoter regions of genes, however, they can also be located within gene bodies (58). Promoter methylation is generally associated with transcriptional repression, while gene body methylation is associated with enhanced transcription (60). Promoter methylation hampers gene expression mainly via two mechanisms: (i) by fostering transcriptional silencing, or (ii) by preventing the recruitment of transcription factors (61). Specifically, methylated cytosines are recognized by DNA methyl-binding proteins (MBPs) that repress gene transcription by preventing the interaction of transcription factors with the promoter (62). Alternatively, DNA methylation may recruit specific proteins that may also favor the recruitment of enzymes catalysing histone posttranslational modifications (PTMs) with subsequent gene repression (63, 64).

DNA methylation is a relatively stable epigenetic signature, it can be tissue-specific and, most importantly, it can be transmitted to the offspring, a phenomenon known as “epigenetic inheritance” (65). Different families of enzymes, known as methyltransferases (DNMTs), are involved in the regulation of DNA methylation: DNMT1 is responsible for the maintenance of methylation patterns in the genome by replicating the hemi-methylated CpG sites (66), whereas Dnmt3a/b are considered de novo methyltransferases (67). Methylation of DNA is a dynamic and reversible process governed by methyl-writing and -erasing enzymes (58). DNA demethylation can be achieved by either passive or active mechanisms (58). Active DNA demethylation consists in the removal of the methyl group by breaking a carbon–carbon bond. DNA demethylation may follow two main pathways: the first is dependent on cytosine deamination (AID, APOBEC3G, FTO) while the second is dependent on the oxidation of methylated cytosines (68). This latter reaction is catalysed by members of the Ten-eleven translocation (TET) proteins family (TET1-3) that convert 5-methylcytosine (5mC) into 5-hydroxymethylcytosine (5hmC) (69, 70). TET1 is mostly found in embryonic stem cells, whereas TET2 and TET3 are ubiquitously expressed. TET1- 3 proteins could further oxidize 5hmC to 5-formylcytosine (5fC) and 5-carboxylcytosine (5caC) that are recognized and excised by the thymine DNA glycosylase (TDG) via the base excision repair pathway (70, 71). By contrast, passive DNA demethylation is the result of DNMT1 inhibition during DNA replication (69).

### Histone Modifications

DNA is packaged into repeating units called nucleosomes by wrapping around multimeric histone proteins. When nucleosomes are organized into tightly packed bundles (heterochromatin), the transcriptional machinery is hampered by a reduction of chromatin accessibility. Conversely, when chromatin is relaxed (euchromatin), DNA is more accessible to transcription factors, and gene transcription may occur (72). Histones are amenable to many posttranslational modifications (PTMs), which include methylation, acetylation, ubiquitination, phosphorylation, SUMOylation, GlcNAcylation, carbonylation, and ADP-ribosylation (73, 74). Of interest, these modifications may cluster in different patterns to regulate chromatin accessibility (59, 72, 75). Albeit the biological significance of many PTMs remains to be elucidated, considerable advances have been made in the understanding of lysine acetylation and methylation (74).

Histone acetylation, characterized by the addition of positively charged acetyl groups to amino acid residues at the level of histone tails, reduces the affinity of histones for DNA thus increasing chromatin accessibility (76). Acetylation occurs mainly on lysine residues on histones H3 and H4; this mark mainly associates with activation of transcription by enhancing chromatin accessibility (77). In this context, bromodomain and extra-terminal proteins recognize histone acetylation marks and initiate the assembly of the transcriptional machinery (78). By contrast, non-acetylated histones have been observed in transcriptionally silent genes where chromatin is compact (79). Acetylation is modulated by histone acetyltransferases (HATs) and histone deacetylases (HDACs) which are involved in addition or removal of an acetyl group, respectively (80). This modification is driven by recognition and binding of transcription factors able to recruit one of a growing family of HATs, namely CBP/p300, MYST, and GNAT (59, 73). HATs catalyse the addition of two-carbon acetyl groups to lysine residues from acetyl-CoA thus leading to gene expression (81). On the other hand, removal of acetyl groups from histone residues by HDACs represses gene transcription (82, 83). Several HDACs have been reported in humans, and they are subdivided into four classes (Class I, IIa, IIb, III, and IV) (84, 85).

In contrast to lysine acetylation, which enhances gene expression, histone methylation may result in different chromatin states according to the methylated residue and the number of added methyl groups (79). Histone methylation is defined as the transfer of methyl group from S-adenosyl-L-methionine to lysine or arginine residues of histone proteins by histone methyltransferases (HMTs) (86). Histone methyltransferases (HMTs) have higher specificity as compared to HATs (87) and include several families of enzymes (EZH, SETD, PRDM, PRMT, METTL, and MLL) (88). Recent evidence indicates that a fine balance between histone methylation and demethylation plays a pivotal role in the regulation of chromatin accessibility.

Several lysine demethylases specific for diverse histone lysine residues have been identified (89). HDMs include members of UTX/Y, JARID1, JMJD, LSD, PHF, and FBXL enzyme families (88).

Interestingly, modifications of histones may reciprocally influence or eventually affect DNA methylation (74). In this regard, recent evidence suggests that DNA methylase (DNMTs), histone methyltransferase (HMTs), and histone acetyltransferase (HATs) are closely interconnected to regulate chromatin remodeling under specific stimuli (90). A well-described crosstalk between DNA methylation and histone H3K9 methylation, mediated by the heterochromatin protein 1 (HP1), represents a valid example of how histone modifications may facilitate the recruitment of enzymes (DNTM3a/b) involved in DNA methylation (91). Another example is methyl-CpG binding protein 2 (MECP2), which recruits the histone methyltransferase SUV39H1 only after binding methylated DNA (92, 93). Therefore, chromatin modifications may influence each other and can propagate.

## Epigenetic Remodeling of Mitochondrial DNA

Increasing evidence suggests that aberrant mitochondrial DNA (mtDNA) modification play an important role in disease development and progression (94). Since the vast majority of mitochondrial proteins are encoded in the nuclear genome, appropriate communication between the nuclear, cytoplasmic and mitochondrial compartments is essential for maintaining proper mitochondrial function. The mitochondrial genome consists of roughly 1,500 genes distributed across the maternal mtDNA and nuclear DNA (nDNA) (95). Human mtDNA is a 16.5-kb circular double-stranded DNA containing a heavy (H) and a light (L) strand located in the mitochondrial matrix (96, 97). mtDNA forms an mtDNA–protein complex, known as nucleoid, with a range of proteins including prohibitins, ATPase family AAA domain-containing protein 3 (ATAD3), mitochondrial transcription factor A (TFAM) and POLG (DNA polymerase gamma, catalytic subunit) (98, 99). In contrast to nDNA, human mtDNA is maternally inherited, is intronless, and lacks histones (100). It contains 37 genes encoding 13 subunit of the oxidative phosphorylation (OXPHOS) complexes I, III, IV, and V; two rRNAs; and 22 tRNAs (2). All other mitochondrial proteins, including those required for mtDNA replication and transcription, are encoded in the nucleus and translocated to the mitochondria using specialized import systems which often involve N-terminal mitochondrial targeting sequences (101).

**Figure 3:**
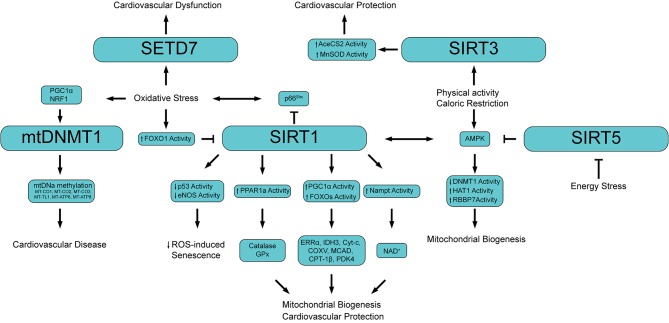
Schematic showing the main epigenetic networks regulating mitochondrial functionality and cardiovascular disease. PGC1α, Peroxisome proliferator-activated receptor gamma coactivator 1-alpha; NRF1, Nuclear respiratory factor 1; mtDNMT1, Mitochondrial DNA methyltransferase 1; MT-CO (1-3), Cytochrome c oxidase subunit (I, II, III); MT-TL1, Mitochondrially encoded tRNA-Leu 1; MT-ATP (6, 8) Mitochondrially encoded ATP synthase membrane (subunit 6, 8); FOXO1, Forkhead box O3; PPAR1a, Peroxisome proliferator-activated receptor 1 alpha; GPx, Glutathione peroxidase; ERRα, Estrogen-related receptor alpha; IDH3, isocitrate dehydrogenase 3; Cyt-c, Cytochrome c; COXV, Cytochrome c oxidase subunit 5; MCAD, Medium-chain acyl-CoA dehydrogenase; CPT-1β, Carnitine Palmitoyltransferase 1 beta; PDK4, Pyruvate dehydrogenase lipoamide kinase isozyme 4; Nampt, Nicotinamide phosphoribosyltransferase; NAD^+^, Nicotinamide adenine dinucleotide; AceCS2, acetyl-CoA synthetase 2; AMPK, 5′ adenosine monophosphate-activated protein kinase; HAT1, Histone acetyltransferase 1; RBBP7, Retinoblastoma binding protein 7.

Emerging evidence suggests that mtDNA may also be regulated at the epigenetic level in the form of mtDNA methylation (2). While nDNA methylation is a well-established feature, mtDNA methylation has been a matter of debate (94, 102). The prevailing opinion was that mtDNA cannot be methylated for two main reasons: (i) methylase cannot access mitochondria, and (ii) mtDNA is not complexed with histones (103). Only recently, mtDNA has been reported to contain 5-methylcytosine (5mC) as well as 5-hydroxymethylcytosine (5hmC) at CpG dinucleotides. In 2011, Shock et al. have identified a mitochondrially targeted DNMT1 transcript variant (mtDNMT1) that uses an upstream alternative translation start site leading to the inclusion of a mitochondrial targeting sequence (101). mtDNMT1 binds to the mitochondrial genome in a manner proportional to the density of CpG dinucleotides. Of note, cytosine methylation in mtDNA may play different role. Indeed, mtDNA methylation represses gene expression from the light-strand promoter. However, increased or no change in transcription of genes from the heavy-strand promoter raises the possibility of a different mode of action (104). This DNMT1 variant is upregulated by the hypoxia-responsive transcription factors peroxisome proliferator-activated receptor gamma coactivator 1 alpha (PGC1a) and nuclear respiratory factor 1 (NRF1) suggesting a regulatory role of mtDNMT1 during vascular oxidative stress (Figure 3) (101).

Besides mtDNMT1, no other specific mitochondrially targeted isoforms of enzymes involved in DNA methylation or hydroxymethylation are known (100). Nevertheless, other enzymes, namely DNMT3A/B and ten–eleven translocation (TET) 1 and 2, have been detected in the mitochondrial protein fraction (105). Interestingly, the presence of these enzymes in the mitochondria seems to be tissue specific. Indeed, inside the mitochondria of ‘excitable tissues’ (heart, skeletal muscle, and neurons) only DNMT3A but not DNMT3b has been detected (106). Furthermore, epigenetic modifications of mtDNA can modulate the activity of nDNA, and vice versa (107). Under conditions of oxidative stress, such as exposure to hypoxia or ethanol, DNMT1 is upregulated and suppresses the expression of ND6 (101), while ND1 is upregulated. Although the significance of opposite ND1 and ND6 regulation is poorly understood, a proposed mechanism involves the interaction of MTERF1 (mitochondrial terminator factor 1) with 5-methylcytosine in the CpG dinucleotides and/or its interaction with mtDNA-bound mtDNMT1 (94).

An interesting study by Byun et al. showed a higher mtDNA methylation level in workers highly exposed to airborne pollutants compared to low airborne pollutant exposed subjects (108). In line with this finding, in a cohort of 81 individuals aged 18-91, methylation levels of the mitochondria gene 12S rRNA inversely correlated with age suggesting that mtDNA methylation may represent an epigenetic marker of ageing (109). In the retina of diabetic mice mtDNA methylation was found associated with mtDNA damage characterized by increased base mismatches and hypermethylated cytosines. Interestingly, inhibition of DNA methylation, or regulation of cytosine deamination, attenuated base mismatches at the D-loop thus preventing mitochondrial dysfunction and microvascular damage. In this study epigenetic signals of mtDNA were driven by oxidative stress as overexpression of Sod2 was able to prevent diabetes-induced D-loop hypermethylation and increase in base mismatches (110). Of clinical relevance, retinal microvasculature from human donors with diabetic retinopathy presented similar increase in D-loop methylation and decrease in mtDNA transcription (111). In another study, analysis of mtDNA methylation by bisulfite sequencing in senescent endothelial cells showed alteration in the methylation pattern of several genes regulating mitochondrial function and metabolism (112). Patients with cardiovascular disease display a significantly higher mtDNA methylation of genes encoding for cytochrome *c* oxidases (MT-CO1, MT-CO2, MT-CO3), tRNA leucine 1 (MT-TL1) and (1.67%, *P* = 0.0001) as well as genes involved in ATP synthesis (MT-ATP6 and MT-ATP8) (113). The latter study suggests that mtDNA methylation could serve as non-invasive and easy-to-obtain epigenetic biomarker and may be implicated in the etiology of CVD (Figure 3).

## Histone Post-Translational Modifications and Mitochondrial Function

Growing evidence indicates that PTMs of histones, mainly at lysine and arginine residues, significantly affect chromatin accessibility thus enabling cell-specific transcriptional programs implicated in mitochondrial dysfunction and vascular disease (Figure 3). Sirtuins are class III histone deacetylases (HDACs), homologs of the yeast protein Silent Information Regulatory 2 (Sir2), a deacetylase involved in yeast metabolism and lifespan (104). The sirtuin family of deacetylases include seven enzymes differentially distributed throughout the cell: SIRT1 and SIRT2 which are mainly localized in both cytoplasmic and nuclear compartments; SIRT3 SIRT4, and SIRT5, which are localized in the mitochondria, and SIRT6 and SIRT7 which are located in the cell nucleus (29, 114, 115). The deacetylation reaction catalysed by sirtuins is NAD+-dependent, and leads to the formation of *O*-acetyl-ADP ribose (AADPR) which can be used as a donor group in ADP-ribosylation reactions (116). In term of activity, all the above-mentioned sirtuins display a deacetylase activity with the exception of SIRT4 which is mostly an ADP-ribosyl transferase, and SIRT6 which exhibits both activities (104).

Available evidence indicates that sirtuins act as pivotal regulators of life span and life-extending effects of calorie restriction (2). Among the different sirtuins, SIRT3 is particularly active in the mitochondria, where it is responsible for the deacetylation of the acetyl-CoA synthase enzyme (AceCS2) (117, 118). Under appropriate nutritional conditions, AceCS2 is completely inactivated upon acetylation at Lys-642, while it is rapidly reactivated by SIRT3 deacetylation (117). Deacetylation of AceCS2 by SIRT3 increases AceCS2 activity leading to the formation of O-acetyl-ADP-ribose and nicotinamide (118), important metabolites implicated in biosynthetic and regulatory purposes (119). In line with these studies, genetic deletion of SIRT3 in mice or gene downregulation as the result of high fat diet feeding, are associated with early metabolic abnormalities which are mainly the result of mitochondrial dysfunction (120). SIRT3 also regulates mitochondrial oxidative stress levels by deacetylation of the antioxidant enzyme MnSOD (121). Although not localized in the mitochondria, SIRT1 is a major regulator of mitochondrial function via deacetylation of PGC1α and FOXOs proteins (122). SIRT1-mediated activation of these target proteins leads to increased mitochondrial respiration and lipid oxidation through regulation of several genes (i.e., ERRα, IDH3, Cyt-c, COXV, MCAD, CPT-1β, and PDK4) required in energy-depleted cell (29, 123). SIRT1 is also critically involved in a dynamic cross-talk with AMPK, a key molecular effector involved in cellular metabolism. Activation of SIRT1/AMPK by physical activity or caloric restriction is associated with an increased usage of lipids as an energy source, mitochondrial biogenesis as well as with an increased expression of nicotinamide phosphoribosyl-transferase (Nampt), the rate-limiting enzyme in NAD+ bio-synthesis. The increase in Nampt activity leads to higher NAD+ production, which in turn activates SIRT1 (124).

Of note, activation of AMPK by SIRT1 seems to be particularly important for the phosphorylation of three main proteins involved in epigenetic remodeling: the DNA methyltransferase DNMT1, the histone acetyltransferase HAT1, and RBBP7, which inhibits DNMT1 and is a HAT1 coactivator (125). AMPK-mediated phosphorylation of these proteins triggered nucleosome remodeling thus favoring the transcription of nuclear-encoded genes involved in mitochondrial biogenesis and function (125). These results show that SIRT1-AMPK axis coordinates mitochondrial function with energy status through epigenetic regulation of nuclear gene expression. SIRT1 is also highly sensitive to the cellular redox state, and confers cardioprotection by counteracting oxidative stress through deacetylation of multiple cellular targets (126–128). In the human endothelium, SIRT1 antagonizes H_2_O_2_-induced premature senescence through its negative modulation of p53 by deacetylation of Lys-373, Lys-382, and Lys-320 (129). Conversely, endothelial SIRT1 overexpression reversed oxidative stress-induced premature senescence through activation of endothelial nitric oxide synthase (eNOS) (130). SIRT1 has also shown to deacetylate FOXO3 thus preventing cellular apoptosis via a mechanism involving the tumor suppressor p53 (131, 132). On the other hand, ROS-dependent acetylation of FOXO1 inhibits its transcriptional activity on SIRT1, catalase (CAT), and MnSOD target genes thus creating a detrimental vicious cycle driven by oxidative stress (133). This molecular circuitry is reinforced by the activation of the mitochondrial adaptor p66^Shc^ which further amplifies ROS levels (134). Interestingly, SIRT1 controls mitochondrial oxidative stress by regulating the transcription of p66^Shc^ (135–137). SIRT1-dependent deacetylation of histone 3 reduces chromatin accessibility on p66^Shc^ promoter thus impeding transcription. By contrast, SIRT1 downregulation as the results of cardiovascular risk factors induces an open chromatin eventually leading to p66^Shc^ expression, mitochondrial oxidative stress and endothelial dysfunction (138). It has also been shown that SIRT1 overexpression increases mitochondrial biogenesis and expression of antioxidant enzymes, namely catalase and glutathione peroxidase (GPx), via activation of the peroxisome proliferator-activated receptor coactivator (PPAR) 1-a activation (139).

SIRT5, a weak deacetylase with strong desuccinylase, demalonylase, and deglutarylase activity, has been also implicated in regulating different aspects of mitochondrial metabolism and cardiovascular function (140). SIRT5 downregulation was recently associated with mitochondrial dysfunction in endothelial progenitor cells of patients with arterial hypertension (141). Other studies reported that SIRT5 deficiency exert a protective role by suppressing mitochondrial ATP production and promoting AMPK activation in response to energy stress. Moreover, genetic deletion of SIRT5 protects against ischemic stroke via modulation of PI3K/Akt pathway (142).

Recent evidence suggests that in the diseased aorta containing atherosclerotic plaques and grafted arteriosclerosis, REF1/H3K9me3 pathway is suppressed thus leading to an increase in the mitochondrial translocation of the AIP1B isoform with subsequent generation of mitochondrial ROS and EC activation (143).

## Mitochondrial ROS and Epigenetic Changes

Mitochondrial-generated ROS have a major impact on DNA methylation. ROS can directly convert 5-methylcytosin (5mC) to 5-hydroxymethylcytosine (5hmC) which blocks the activity of DNMT1 leading to an improper methylation inheritance during mitosis and global hypomethylation (144). Moreover ROS can oxidize guanosine to 8-oxo-20-deoxyguanosine (8-oxodG) thus inhibiting methylation of adjacent cytosine and further contributing to global hypomethylation of DNA (145, 146). The formation of 8-oxodG in particular loci promotes the transcription of pro-inflammatory genes in response to TNF-α (147). Furthermore, 8-oxodG interacts with HIF1α thus affecting its ability to bind VEGF promoter with subsequent impairment of angiogenesis (148). In line with these observations, two recent meta-analyses showed that high levels of 8-oxodG are associated with atherosclerotic vascular disease and predict outcome (149, 150). High ROS levels also influence both repressive (H3K9me2/3 and H3K27me3) and active histone marks (H3K4me2/3) (151, 152).

Similarly to DNA methylation, histone methylation is dependent on SAM availability and is therefore reduced in the presence of high ROS levels (153, 154). In support of this hypothesis in a model of cardiac pressure overload the SET and MYND domain containing protein 1 (SMYD1) methyltransferase was significantly downregulated (155). On the other hand, several studies showed that hyperglycemia-induced oxidative stress increases the expression of the methyltransferase SETD7 and its epigenetic marker H3K4m eventually leading to enhanced transcription of inflammatory and oxidant genes, thus generating a vicious cycle (Figure 3) (156).

## Mitochondrial Metabolites as Cofactors for Chromatin Modifications

**Figure 4:**
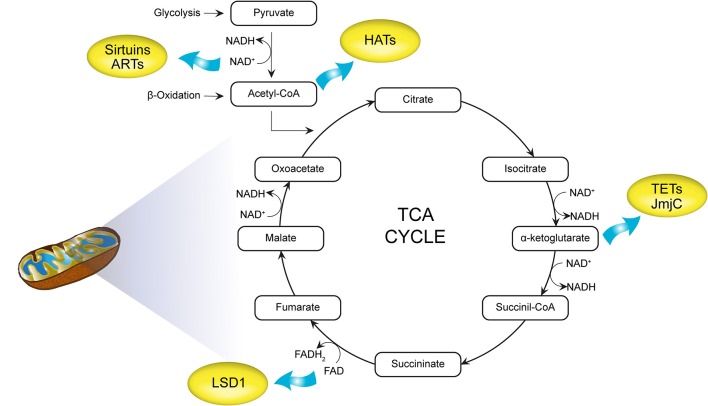
Intermediate mitochondrial metabolites as cofactors for chromatin modifications. acetyl-CoA generated by glycolysis and β-oxidation acts as a substrate for histone acetyltransferases (HATs). Nicotinamide adenine dinucleotide (NAD^+^) is required for histone deacetylases (HDACs; histone deacetylation) as well as ADP-ribosyltransferases (ARTs). α-Ketoglutarate and flavin adenine dinucleotide (FAD+) are cofactors for DNA (ten-eleven translocations, TETs) and histone demethylases [Jumonji C domain containing (JmjC), LSD1]. TCA, tricarboxylic acid cycle; HATs, histone acetyltransferases; NAD^+^, nicotinamide adenine dinucleotide; HDACs, histone deacetylases; ARTs, ADP-ribosyltransferases; FAD, flavin adenine dinucleotide; TETs, ten-eleven translocations.

By serving as essential cofactors for most chromatin-modifying enzymes, important intermediates of cell metabolism and dietary intake allow the integration of metabolic information and transcriptional control (Figure 4). Fluctuating metabolite concentrations are therefore proposed to provide signalling cues for continual adjustment of gene expression by modulating the epigenome to influence chromatin dynamics. Additional biochemical evidence suggests that energy metabolite concentration could affect PTMs of the chromatin-modifying machinery itself, in turn regulating enzymatic activity, stability, and chromatin binding capacity associated with gene expression (54).

### NAD+

NAD+ is an essential cofactor for reactions catalysed by the highly conserved SIRT HDAC family (2). Other NAD+ consuming enzymes such as ADP-ribosyltransferases have also been shown to covalently ADP-ribosylate core histones (157). PAR polymerases (PARPs) utilize NAD+ to catalyse poly(ADPribose) synthesis and are involved in the cellular stress response (158). Poly(ADP-ribose) polymerase-1 (PARP1), a major member of the PARP family, is a nuclear protein involved in chromatin remodeling and promotion of DNA repair (159). However, several studies report that in condition of oxidative stress PARP-1 also localizes to mitochondria (160–162). Mitochondrial PARP-1 is reported to actively participate in maintenance of functional integrity of the organelles (163) and to play a detrimental role when hyperactivated (160, 164). Furthermore, the potential role of PARP1 as a nuclear epigenetic regulator for the maintenance of mitochondrial DNA integrity has been suggested (159). Indeed, PARP-1 suppression reduces mtDNA integrity, as well as the expression of mitochondria-encoded respiratory complex subunits COX-1, COX-2, and ND-2 (164). Accordingly, PARP-1 localizes at promoters of nuclear genes encoding both the mtDNA repair proteins UNG1, MYH1, and APE1 and the mtDNA transcription factors TFB1M and TFB2M (164). Consistent with these findings, PARP-1 suppression impairs mitochondrial ATP production (164).

### S-Adenosylmethionine

S-Adenosylmethionine (SAM) is produced by the condensation of methionine and ATP during the first of nine steps required for the conversion of methionine to succinyl-CoA, a predominantly cytoplasmic pathway that ends up in the mitochondria (29). It contains the active methyl-donor group utilized by most methyltransferase enzymes. It has been demonstrated that ROS can reduce SAM availability, thus limiting the activity of DNA and histone methyltransferases (145). This is achieved either by inhibiting methionine adenosyl-transferase and thus SAM synthesis or by inhibiting methionine synthase and thus methionine regeneration (56). Interestingly, long-term exposure to H_2_O_2_ decreased SAM levels leading to hypomethylation of the long interspersed nuclear element-1 (LINE-1) (165). LINE-1 hypomethylation as an indicator of global methylation status was found in blood from patients with ischaemic heart disease and stroke, and has been related to higher risk for these diseases (166).

### FAD+

Derived from the vitamin riboflavin (vitamin B2), mitochondrial-generated FAD functions as the prosthetic group for certain oxidation–reduction enzymes (2). For example, LSD1 demethylase is a FAD+-dependent enzyme capable of demethylating H3K4me1/2 and H3K9me1/2 (167). LSD1 activity is regulated by redox state and it is stimulated when FAD is oxidized (168). LSD1, in turn, regulates mitochondrial respiration and energy expenditure. Specifically, LSD1 binds directly to genes such as PGC1α, PDK4, FATP1, and adipose triacylglycerol lipase (ATGL), and represses their transcription associated with loss of H3K4 methylation (169).

### β-Hydroxybutyrate

The ketone body β-hydroxybutyrate (βOHB) modulates several signalling pathways with implications for metabolic disease and diabetes (170). Prolonged fasting, calorie restriction, strenuous exercise, or ketogenic diets are conditions associated with increases in serum concentrations β-OHB (171). Interestingly, βOHB is an endogenous inhibitor of many NAD+-independent HDACs (172). HDAC inhibition by βOHB might affect the pathogenesis of type 2 diabetes in at least two ways: through direct regulation of HDAC-dependent glucose metabolism, or by promoting resistance to oxidative stress (170). For examples, βOHB-mediated inhibition of HDAC1 and HDAC2 increases acetylation of histone H3K9 and H3K14 and establishes a permissive chromatin configuration for the expression of Foxo3 with subsequent transcription of its downstream antioxidant genes such as catalase and MnSOD (172). Similarly, βOHB may have similar effects on mitochondrial function, glucose homeostasis, and obesity through endogenous inhibition of HDAC3. The mechanism for these metabolic benefits of class I HDAC inhibition may be the upregulation of PGC1α in a variety of tissues (173, 174). Transcription of FGF21 is similarly upregulated via βOHB-mediated inhibition of HDAC3 which results in the activation of ketogenesis in obese mice (175). The microvascular and macrovascular complications of type 2 diabetes are thought to be due in part to increased oxidative stress brought on through several pathways including polyols, protein kinase C, hexosamine, and advanced glycosylation end products (176). In this context, the emerging role of βOHB in suppressing oxidative stress may be relevant for the management of diabetic complications. Other studies have previously suggested a role for both βOHB and HDAC inhibitors in the protection from oxidative or ischemic stress (170).

### α-Ketoglutarate

Connections between metabolic cofactors and enzymes associated with the removal of epigenetic methyl modifications are also emerging (54). The TET family of dioxygenases mediate the oxidation of 5mC. The potential for the TET family (TET1/2/3) to regulate diverse physiological functions including metabolic signalling requires the TCA cycle metabolite α-KG, and this activity is inhibited by 2-hydroxyglutarate (2HG) (2) (Figure 4). This means that oxygen deficiency and disturbances in mitochondrial metabolism could affect the activation of TET enzymes and thus control DNA methylation (177). Hearts of mice exposed to high-fat diet (HFD) showed reduced levels of αKG and this observation was paralleled by a compromised TET1 function. Accordingly, an exogenous source of αKG restored the DNA demethylation cycle, glucose uptake, and insulin response (178).

Jumonji C domain-containing histone demethylases are α-KG-dependent (177). Although studies are yet to determine the TET-metabolism connection, mutations in isocitrate dehydrogenase genes are associated with reduced α-KG and elevated 2HG levels leading to genome-wide changes in histone and DNA methylation patterns (54).

The Jumonji C domain (JmjC) containing lysine demethylases (KDM) are the largest group, which can be divided to six subgroups (KDM2-7) depending on their chromatin interacting domains and substrate specificity (179). The activation of these enzymes is also dependent on the presence of α-KG. Therefore, disturbances in Krebs cycle function can affect histone methylation and gene expression (177).

### Acetyl-CoA

Acetyl-CoA generated from glucose and fatty acid metabolism feeds into the TCA cycle to contribute to cellular energy supply. Importantly, acetyl-CoA is the essential acetyl group donor to lysine acetylation reactions and both pharmacological and genetic interventions that modify cellular acetyl-CoA concentrations directly affect acetylated proteins including histones (180). Because histone acetylation is ubiquitously associated with open chromatin and gene expression, acetyl-CoA links intermediary carbon metabolism with chromatin dynamics and transcription (54).

## Epigenetic Therapies

Targeting epigenetic modifications is a highly promising approach to restore gene expression and to rescue or prevent mitochondrial insufficiency and vascular dysfunction. There are several examples of how specific interventions can be employed to modify the landscape of DNA/histone modifications in this setting.

Studies in knockout mice have shown that class I HDACs play a key role in regulating metabolism. Chronic treatment with butyrate, a broad HDAC inhibitor that is expected to phenocopy HDAC3 loss-of-function, prevents metabolic alterations in diet-induced obese as well as in aged mice, mainly by enhancing oxidative phosphorylation and beta-oxidation in mitochondria (181, 182). Butyrate treatment also improves mitochondrial biogenesis via epigenetic modulation of PGC-1α as well as induction of several microRNAs such as miR-133a-3p, miR-208b, and miR-499-5p, implicated in the regulation of mitochondrial potential and integrity (183). Similarly, the class I HDAC inhibitor SAHA, but not a class II HDAC inhibitor, increases the expression of PGC-1α thus leading to enhanced mitochondrial biogenesis, oxygen consumption in adipose tissue and skeletal muscle from mice with type 2 diabetes (174). These changes were associated with a significant improvement of insulin sensitivity, metabolic rate and oxidative metabolism (174). Moreover, treatment with SAHA was also found to reduce ischemia-reperfusion injury following myocardial infarction and to prevent apoptosis in cultured myocytes subjected to hypoxia/reoxygenation (184, 185).

Pharmacological modulation of sirtuins has also shown to impact on mitochondrial functionality and vascular function (186). Although primarily known as a nuclear protein, SIRT1-mediated deacetylation of PGC-1α has been extensively implicated in metabolic control and mitochondrial biogenesis, which was proposed to partially underline SIRT1 role in caloric restriction and impacts on longevity. Moreover, recent evidence suggests that modulation of SIRT1 activity may also affect the turnover of defective mitochondria by mitophagy (187). In line with these evidences, SIRT1 activation by resveratrol improves vascular function while attenuating dyslipidaemia and obesity-induced metabolic alterations in human subjects (188). SIRT1-dependent improvement of flow-mediated dilation can be partially explained by increased deacetylation of p66^Shc^ promoter as well as posttranslational and transcriptional regulation of endothelial NO synthase (eNOS) (137, 189). Indeed, SIRT1 inhibition significantly increases p66^Shc^ transcription, mitochondrial oxidative stress and organelle disruption. Whereas, in both the diabetic vasculature and myocardium activation of SIRT1 suppresses p66^Shc^ signalling thus preventing the accumulation of H_2_O_2_ in mitochondria and cellular death (137, 138, 190). Pharmacological activation of SIRT3 by small molecules, namely 7-hydroxy-3-(4′-methoxyphenyl) coumarin (C12), also represents a promising approach to prevent mitochondrial ROS via deacetylation and activation of MnSOD (121).

Together with SIRT1, other epigenetic modulators participate to the transcriptional regulation of the mitochondrial adaptor p66^Shc^. Modulation of CpG DNA methylation by folates regulates p66^Shc^ transcription (138). Consistently, a recent work found that homocysteine stimulates p66^Shc^ transcription in human endothelial cells via specific CpG dinucleotides demethylation in the p66^Shc^ promoter (191). Of note, p66^Shc^ promoter CpG methylation was significantly reduced in peripheral blood leukocytes of patients with coronary artery disease and high plasma homocysteine levels, thus strengthening the relevance of p66^Shc^-related epigenetic changes in the context of cardiovascular disease (191). Moreover, metformin, a widely used antidiabetic drug, was found to modulate SIRT1-p66^Shc^ signaling in experimental models of diabetes (138, 192, 193).

Inhibitors of histone acetyltransferases have also shown to revert mitochondrial oxidative stress. The dietary compound curcumin, an inhibitor of the histone acetyltransferase CBP/p300, has shown to rescue hyperglycemia-induced endothelial dysfunction by regulating the expression of several pro-oxidant and antioxidant enzymes involved in mitochondrial oxidative stress and mitochondrial biogenesis (194). Similarly, inhibition of another acetyltransferase, GCN5, prevents angiotensin II–mediated downregulation of catalase thus fostering accumulation of mitochondrial ROS (195).

## Conclusions

In conclusion, evidence discussed so far strongly suggests that specific epigenetic signals are responsible for transcriptional changes leading to mitochondrial dysfunction and cardiovascular disease. In turn, the availability of mitochondrial intermediate metabolites controls the activation of chromatin modifying enzymes. The growing understanding of chromatin modifications and their impact on transcription, will open perspective for the development of personalized biomarkers and epigenetic therapies aimed at preventing mitochondrial dysfunction and cardiovascular disease.

## Author Contributions

SM, SA, FP, and SC drafted the manuscript and prepared the graphical illustrations. TL revised the manuscript and figures.

### Conflict of Interest

The authors declare that the research was conducted in the absence of any commercial or financial relationships that could be construed as a potential conflict of interest. The handling editor declared a past co-authorship with one of the authors FP.
